# Physical activity and sleep differences between osteoarthritis, rheumatoid arthritis and non-arthritic people in China: objective versus self report comparisons

**DOI:** 10.1186/s12889-021-11837-y

**Published:** 2021-10-09

**Authors:** Ting Xu, Xiaojun Jia, Shuanghong Chen, Yingying Xie, K. K. Tong, Tony Iezzi, Todd Jackson

**Affiliations:** 1 Experimental Health Psychology, Clinical Psychological Science, Faculty of Psychology and Neuroscience, Maastricht University, Maastricht, Netherlands; 2 Key Laboratory of Cognition and Personality (Ministry of Education), Southwest University, Chongqing, 400715 China; 3 Department of Psychology, University of Macau, Macau, 000978 Taipa S.A.R. China; 4 Department of Psychology, London Health Sciences Centre, London, ON 97403 Canada

**Keywords:** Physical activity, Sleep, Osteoarthritis, Rheumatoid arthritis, Sensewear, Questionnaires, Accelerometer

## Abstract

**Background:**

Objectively measured differences in physical activity (PA) and sleep have been documented among people with osteoarthritis (OA) and rheumatoid arthritis (RA) compared to non-arthritic controls. However, it is not clear whether OA and RA subgroups also differ on these indexes or the extent to which distinct arthritis subgroups versus controls can be accurately identified on the basis of objective PA and sleep indexes compared to self-report responses on questionnaires. This study addressed these gaps.

**Methods:**

This case-control study comprised Chinese adults with OA (*N* = 40) or RA (*N* = 40) diagnoses based on physician assessments as well as a control group of adults without chronic pain (*N* = 40). All participants wore a Sensewear Armband (SWA) for consecutive 7 days and completed the International Physical Activity Questionnaire Short Form-Chinese as well as Pittsburgh Sleep Diary to obtain objective and subjective PA and sleep data, respectively.

**Results:**

There were no differences between the three groups on any self-report indexes of PA or sleep. Conversely, OA and RA subgroups displayed significantly lower PA levels and more sleep problems than controls did on a majority of SWA indexes, though arthritis subgroups were not differentiated from one another on these measures. Logistic regression analyses indicated four non-multicollinear SWA indexes (i.e., steps, active energy expenditure, vigorous activity, time awake after sleep onset) correctly identified the subgroup membership of 75.0–82.5% of participants with RA or OA while classification accuracy results were attenuated for controls.

**Conclusions:**

Where possible, objective measures should be used to assess PA and sleep of adults with OA and RA while particular self-report PA questionnaires should be used sparingly.

**Supplementary Information:**

The online version contains supplementary material available at 10.1186/s12889-021-11837-y.

## Background

Deficits in physical activity (PA) and sleep are prevalent within arthritis populations [1–6], contribute to reduced health-related quality of life (HRQoL) [7–9] and predict poor health outcomes such as cardiovascular disease morbidity and mortality [10, 11]. These data have been derived from objective measures of PA and sleep (e.g., accelerometers) as well as complementary self-report questionnaires. Although questionnaires are inexpensive and convenient to use, PA and sleep estimates calculated from them correlate inconsistently with objective indexes [12–14], and appear to discriminate arthritis samples from controls less accurately than objective measures do [15, 16].

Accelerometers have emerged as valid objective alternatives to self-report responses from questionnaires and assess PA in contexts of daily life [17]. Small, unobtrusive, comfortable to wear devices can track intensity, duration, and frequency of PA in a manner that controls for potential biases in recall and social desirability. Some PA trackers such as the Sensewear Armband (SWA) have added advantages of differentiating sedentary activity from sleep and generating accurate data regarding sleep parameters [16].

Studies based on objective assessments have documented less frequent, less intense PA levels and/or more frequent sleep disturbances within various arthritis subgroups compared to non-arthritic controls [16–19]. To illustrate, Prioreschi et al. [17] used accelerometers to assess habitual PA of rheumatoid arthritis (RA) patients and non-arthritic controls. RA patients displayed significantly more sedentary activity than controls did. Higher PA levels were also related to better HRQoL. In other research, osteoarthritis (OA) patients and non-arthritic controls showed no significant differences in average daily energy expenditures but the former group displayed less PA based on average steps per day [18].

Despite evidence of arthritis versus control differences on objectively assessed PA and sleep, the associated literature has important gaps. First, arthritis subgroup (e.g., OA vs. RA) comparisons of PA and sleep have received comparatively little attention. Studies have reported no differences between patients with RA versus fibromyalgia [5] or lupus [15] but may have been underpowered due to small sample N’s. Evaluations within larger samples would provide more rigorous tests of arthritis subtype differences in PA and sleep. In addition, at least with respect to sleep, most studies have been conducted in samples with fibromyalgia or inflammatory diseases including RA so comparatively little is known about the nature or severity of sleep disturbances in OA samples [20]. More fully evaluating arthritis subgroup similarities and differences in sleep and PA may provide foundations for examining the extent to which specific interventions designed to target particular deficits in sleep or PA have utility within particular RA versus OA patient subgroups. Furthermore, because evidence is based almost exclusively on samples from Western countries, it is not clear whether findings apply to groups in understudied yet highly populated low-and middle-income countries. To illustrate, overall rates of OA and RA in China are comparable to or higher than those reported in higher income Western countries [21, 22] but the relative paucity of well-trained, qualified treatment specialists [23, 24] and low affordability of newer biological agents [25] are more pronounced barriers to care. Clarifying whether and how PA and sleep are affected by arthritis in understudied cultural contexts provides critical foundations for the development and use of informed guidelines to improve these facets of functioning.

This study had two purposes. First, we assessed differences in objective versus questionnaire measures of PA and sleep between adult samples with OA, RA, and non-arthritic controls. Based on related research [15], arthritis subgroups were expected to display comparatively less PA and more sleep disturbances than controls would, especially on objective indexes. Conversely, few OA vs. RA subgroup differences were expected. Second, we assessed the accuracy of significant objective and subjective measures of PA and sleep in identifying membership within each arthritis subgroup versus the control group (i.e., RA group versus controls, OA group versus controls); objective indexes were expected to be more sensitive than self-report questionnaires.

## Method

### Study design

A case-control design was used to assess the physical activity and sleep differences between people with osteoarthritis, rheumatoid arthritis and non-arthritic in China based on objective SWA indexes and self-report questionnaires.

### Participants

The sample comprised Chinese adults with OA (29 women, 11 men) and RA (29 women, 11 men), as well as non-arthritic controls (CON) who did not report ongoing pain (29 women, 11 men).

### Procedure

This study was approved by the Human Research Ethics Committee of Southwest University, Chongqing (534,472,715; Dec., 2018). Participants were recruited via advertisements from local community settings affiliated with the university (i.e., large apartment complexes), two local hospitals, and extended social networks of students (*n* = 9) assisting with data collection. Aside from those who volunteered based on advertisements or hospital contacts, snowball recruiting was used to facilitate data collection which occurred between July and October 2019. Inclusion criteria were (1) age of at least 18 years, (2) a neurologist-based diagnosis of OA, physician diagnosis of RA based on 2010 American College of Rheumatology criteria [26] or the absence of chronic pain lasting 3 months or longer based on participant self-reports (i.e., for non-arthritic controls) and later confirmed by research personnel implementing SWA data collection, and (3) ambulatory independence with minimal assistance (i.e., walking with or without a cane). Exclusion criteria included (1) neurological or psychiatric conditions that could interfere with comprehension, and (2) allergies to copper given the need to wear the armband for extended intervals.

**Fig. 1:**
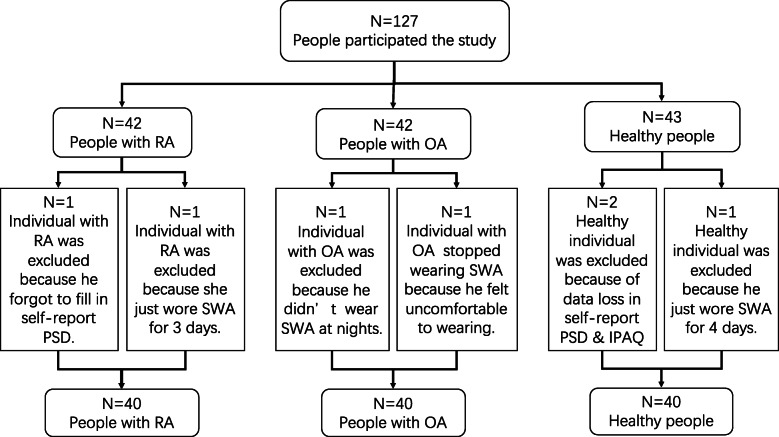
The flow chart of recruitment in the study

Management from contacted settings provided permission to recruit volunteers via print advertisements and contacts from organization staff. Those who wished to be involved were given a general description of the research (i.e., a study on physical activity, sleep and health among adults with arthritis or an absence of ongoing pain), an informed consent detailing the voluntary, confidential nature of participation, estimated time involved (1 week), and compensation (250 yuan), the self-report questionnaires below and an SWA. In addition, based on snowball recruiting, participants could refer interested others they knew who had OA, RA, or did not have arthritis to the research team. Research personnel was on hand to ensure participants met selection criteria and understood data collection procedures in addition to answering queries (see Fig. 1). Although rates of chronic pain including arthritis subtypes tend to be higher among women, at the study outset, we sought to include a minimum of 10 men within each group so that findings would be applicable to both genders and ratios of women to men in each group were similar, hence controlling for potentially confounding effects of gender. Given that non-arthritic participants were easier to recruit than cohorts with arthritis, data collection for controls was completed first (33 women, 11 men). Thereafter, we sought demographically similar arthritis subgroups by matching each arthritis case on gender and approximate age (within 5 years) to a particular control group member having these characteristics.

Participants were asked to wear the SWA for seven consecutive days, except during water-based activities, for at least 21.5 h per day in line with criteria of past work [27]. During the study, participants received a daily text message with reminders to continue wearing the SWA and finish the sleep diary. Sleep diaries were completed every day and the PA questionnaire was completed after SWA data had been collected. Upon completion, each participant was compensated and SWA data printouts were provided and discussed upon request.

### Objective measures

**SenseWear Armband (**SWA; model: MF-SW; Body Media, Pittsburgh, PA, USA).

The SWA is worn over the left tricep and uses multiple sensors to assess heat flux (i.e., heat dissipated from body), galvanic skin response (estimate of skin conductivity) and skin temperature as well as a three-axis accelerometer that estimates energy expenditures at varying metabolic equivalents (METs) from sleeping peacefully to vigorous PA [28]. Aside from these indexes, we assessed sedentary (0–1.5 METs), moderate (3.0–6.0 METs), and vigorous (above 6.0 METs) activity levels, total PA equal to or greater than 3 METs, PA duration, (PAD), number of steps, and time lying down.

SWA reliability and validity have been satisfactory in general samples [29] as well as those with arthritis [28, 30]. Because the SWA differentiates sedentary activity from sleep and is reliable and valid in assessing sleep parameters in various populations [16, 31, 32], data were also collected during the 7 consecutive nights of use. Following Oudegeest-Sander et al. [33], we assessed (1) sleep onset latency (SOL) based on the interval between “lights out” and the beginning of “sleep onset”, (2) waking after sleep onset time (WASO) as duration of “awake” epochs (in minutes) that occurred after sleep onset and before final awakening, (3) total sleep time (TST) as number of minutes sleeping, and sleep efficiency (SE) as total sleep time divided by total lying down time × 100. In this study, intra-class correlations were acceptable across subgroups, with the exception of SOLs in the control group: TST (OA = .90, RA = .82, CON = .86), WASO (OA = .71, RA = .86, CON = .82), SOL (OA = .76, RA = .70, CON = .59), and SE (OA = .90, RA = .87, CON = .87).

### Self-report questionnaires

#### Demographics

Sex, age, height, weight, marital status (no /yes to married or dating), personal education level (from primary school or lower to post-secondary education), and status as a smoker and consumer of alcohol (no versus yes) were assessed. Pain duration in months and pain-related interference with daily activities during the past week rated between *1 = None* and *5 = Extreme* were also assessed in arthritis subgroups.

**International Physical Activity Questionnaire Short Form-Chinese** (IPAQ-SF-C) [34].

The IPAQ-SF is a standardized PA questionnaire that has been used cross-culturally in populations 18–65 years of age [34]. The scale consists of seven items requiring PA estimates during the previous week, including number of days and amount of time spent walking, sitting, or participating in moderate PA (e.g., carrying light loads, bicycling at regular pace, doubles tennis) and vigorous PA (heavy lifting, digging, aerobics, fast bicycling). Acceptable psychometrics have been reported in various population subgroups [34, 35]. Alphas for OA (α = .74) and RA (α = .76) subgroups in this study were satisfactory while that of the CON subgroup (α = .68) approached the conventional threshold of acceptability (α = .70).

**Pittsburgh Sleep Diary** (PSD) [36].

The “waketime” PSD assess several self-reported sleep parameters: (1) SOL in estimated minutes, (2) total time in bed (TIB) based on to bed and wake up times in minutes, (3) frequency of nightly awakenings (FNA) between 0 = not at all and 5 or more = number of awakenings per night, (4) TST from 0 min to TIB, (5) WASO measured as awake minutes after sleep onset before lights on and ranging from 0 min to TIB - SOL – TST, and (6) sleep efficiency percentage (SE) based on the formula, SE = TST / TIB. We also assessed (7) sleep quality (SQ), ranging from 0 = not at all refreshed to 10 = completely refreshed and (8) alertness on final waking, ranging from 0 = not at all alert to 10 = completely alert. Nearly all PSD indexes had acceptable alphas (OA: α = .85 to α = .98, RA: α = .66 to α = .97, CON: α = .79 to α = .96).

#### Data analysis

Group differences in age, BMI, relationship status, education, smoking status, alcohol use status, and pain characteristics were assessed with univariate analyses of variance (ANOVAs) or chi-square tests as appropriate. Multivariate analyses of variance (MANOVAs) evaluated overall group differences on dependent measures of SWA versus questionnaire-assessed PA and sleep. For each set of comparisons, univariate F’s were presented to illustrate group differences on specific indexes; Bonferroni-adjusted post-hoc tests examined specific subgroup differences on measures having significant univariate F values.

To assess multicollinearity between SWA indexes, Pearson correlation coefficients (two-tailed) were initially calculated and measures having bivariate correlations > 0.70 were considered suspect. Furthermore, variance inflation factors (VIFs) were assessed by including an SWA sleep index as the criterion measure and all remaining statistically significant SWA indexes of PA and sleep as predictors within a multiple regression model. Measures with VIF values > 5.0 were treated as multicollinear [37] and excluded from subsequent classification model analyses.

Standard bivariate logistic regression analyses (LGA) assessed accuracies in correctly identifying participant group membership (OA or RA versus control) from responses on non-multicollinear SWA indexes on which significant subgroup differences were found in preceding analyses. Receiver operating characteristic (ROC) curves were generated to identify optimal cutoffs for each unique, significant PA and sleep index that emerged in classification models for RA group versus control group membership or OA group versus control group membership [38].

G*power 3.1.9 was used to calculate the sample size needed to detect an overall (MANOVA) difference in PA and sleep with a medium effect size (i.e., Cohen’s f^2^ = .15 at 90% power with an alpha level of 0.05) based on three groups and 13 dependent variables, per the total number of SWA indexes used in this study. The analysis indicated a minimum N of 108 was necessary to meet this threshold, though we over-sampled in anticipation of an attrition rate of up to 15%, given the high response burdens on participants and seven-day assessment interval.

## Results

### Demographic characteristics of overall sample

On average, participants were 57.22 years of age (SD = 16.64 years) with a majority reporting education of less than high school completion (82%) and a current committed relationship (78%). On average, participants had a body mass index (BMI) of 23.44 (SD = 3.13). Majorities reported neither smoking (83%) nor consuming alcohol (83%) at present.

### Sample differences on demographics

The three groups did not differ on age, BMI, current relationship status or education level. Furthermore, no differences were found for smoking or alcohol consumption status. Finally, arthritis subgroups did not differ on pain duration or pain-related interference with daily activities (see Table 1).

**Table 1:** Sample differences on demographic measures (*N* = 120)

	Group				
	Osteoarthritis (***N*** = 40)	Rheumatoid Arthritis (***N*** = 40)	Control (N = 40)		
Measure	Mean (SD) or N (%)	Mean (SD) or N (%)	Mean (SD) or N (%)	***F*** / *χ*^2^	***p***
Age	57.90 (17.51)	59.35 (16.52)	54.40 (15.87)	0.94	.396
Body mass index	23.73 (3.90)	24.03 (2.59)	22.54 (2.58)	2.62	.077
Current Relationship (Yes)	33 (83%)	27 (68%)	34 (85%)	4.22	.121
Education status				4.03	.855
Primary school	13 (32%)	13 (33%)	14 (35%)		
Middle school	15 (38%)	15 (37%)	18 (45%)		
High school	4 (10%)	4 (10%)	2 (5%)		
Post-Secondary	8 (20%)	4 (20%)	4 (15%)		
Cigarette Smoking status					
Non-smoker	33 (83%)	35 (88%)	31 (78%)	1.39	.500
Smoker	7 (17%)	5 (12%)	9 (22%)		
Current Alcohol Use					
Non-drinker	33 (83%)	32 (80%)	35 (88%)	0.84	.657
Drinker	7 (17%)	8 (20%)	5 (12%)		
Pain Duration (in months)	127.4 (128.26)	139.1 (117.37)	–	0.18	.672
Pain-Related Interference with Daily Activities	3.45 (1.01)	3.50 (1.06)	–	0.05	.830

### Sample differences on objective indexes of physical activity and sleep

A significant multivariate effect was observed for SWA indexes, F (2, 117) = 2.212, *p* = 0.001. Table 2 indicates control group members had significantly less sedentary activity as well as more moderate PA, vigorous PA, overall PA, and active energy expenditures (AEE), longer PA durations, and higher average daily step counts than did cohorts with OA and RA. Arthritis subgroups did not differ from one another on any PA index. On sleep measures, groups did not differ on SOLs or TST. However, controls spent less time awake after sleep onset (WASO) than either arthritis subgroup did and displayed marginally better sleep efficiency than OA subgroup members did (*p* = .053).

**Table 2:** Sample differences on objective indexes of physical activity and sleep

	Group					
	Osteoarthritis (OA)	Rheumatoid Arthritis (RA)	Control (CON)			Post-Hoc
Sensewear Index	M (SD)	M (SD)	M (SD)	***F***	***p***	Comparisons
Physical Activity Index						
Sedentary activity (min/day)	1274.23 (90.91)	1283.10 (93.76)	1207.08 (130.21)	6.10	0.001	CON < OA, RA
Moderate activity (min/day)	148.58 (85.38)	144.43 (93.76)	199.83 (130.21)	3.98	0.021	CON > OA, RA
Vigorous activity (min/day)	3.98 (5.87)	1.95 (3.15)	13.53 (28.81)	5.24	0.007	CON > OA, RA
Total energy expenditure (calories/day)	9485.68 (1808.17)	9426.08 (1728.19)	9918.49 (2520.88)	0.69	0.505	no differences
Active energy expenditure (calories/day)	2544.35 (1578.87)	2439.86 (1660.48)	3854.25 (3226.22)	4.76	0.001	CON > OA, RA
Physical activity level (METs)	1.54 (.24)	1.51 (.27)	1.75 (.32)	8.68	0.001	CON > OA, RA
Physical activity duration (min/day)	152.76 (91.15)	146.74 (97.19)	214.98 (121.37)	5.27	0.006	CON > OA, RA
Steps (No./day)	7767.68 (4002.25)	7230.98 (3861.86)	11,215.54 (4571.00)	10.83	0.001	CON > OA, RA
Lying down (min/day)	534.35 (124.87)	519.59 (104.79)	514.50 (86.26)	0.38	0.688	no differences
Sleep Index						
Sleep onset latency (min/night)	19.63 (13.90)	15.55 (9.46)	14.20 (12.14)	2.23	0.112	no differences
Total sleep time (min/night)	418.50 (78.37)	407.01 (78.20)	405.95 (71.11)	0.34	0.716	no differences
Wake after sleep onset time (min/night)	128.23 (63.99)	116.52 (49.41)	91.76 (51.91)	4.51	0.013	CON < OA, RA
Sleep efficiency (%)	75% (9%)	76% (8%)	80% (9%)	3.02	0.053	no differences

### Sample differences on self-report indexes of physical activity and sleep

For self-report questionnaire measures of PA and sleep, the multivariate F value was not significant, F (2, 117) = 1.088, *p* = 0.359. Furthermore, arthritis subgroups and controls did not differ on any of the individual PA or sleep indexes (see Table 3).

**Table 3:** Sample differences on self-report indexes of physical activity and sleep

Measure	Group			***F***	***p***
	Osteoarthritis (OA)	Rheumatoid Arthritis (RA)	Control (CON)		
	Median (IQR) / M (SD)	Median (IQR) / M (SD)	Median (IQR) / M (SD)		
Physical Activity (IPAQ-SF)^a^					
Sitting (min/week)^a^	1470 (1680)	1575 (1575)	1260 (840)	1.02	.362
Walking (min/week)^a^	1155 (1539)	1270 (1353)	1386 (1905)	0.23	.796
Moderate (min/week)^a^	480 (2235)	480 (2310)	1120 (1860)	0.94	.394
Vigorous (min/week)^a^	0 (540)	0 (690)	0 (1860)	0.18	.838
Total (min/week)^a^	1579.5 (5049)	2206 (5861)	4825 (4684)	0.32	.729
Sleep (PSD)					
Sleep onset latency (min/night)	30.54 (24.22)	30.66 (28.50)	20.50 (14.51)	2.53	.084
Total time in bed (min/night)	480.76 (57.63)	499.87 (87.35)	467.65 (59.23)	2.18	.118
Frequency of nightly awaking	1.89 (0.91)	1.99 (1.10)	1.70 (1.17)	0.77	.464
Total sleep time (min/night)	428.54 (59.60)	429.86 (92.75)	412.75 (59.22)	0.69	.501
Wake after sleep onset time (min/night)	21.68 (37.85)	39.36 (44.88)	32.15 (23.81)	2.36	.099
Sleep efficiency (%)	89.65 (8.82)	86.38 (11.46)	88.5 (5.45)	1.39	.254
Sleep quality (0–10)	7.62 (1.57)	7.36 (1.79)	7.61 (1.60)	0.31	.734
Alertness (0–10)	4.68 (2.46)	5.12 (3.19)	4.50 (2.48)	0.55	.581

^a^ Based on median rather than mean minutes

### Accuracy of Sensewear indexes in identifying group membership

Questionnaire indexes were excluded from LGA due to the absence of group differences in self-reported PA and sleep. Prior to running LGAs, multicollinearity analyses (see Supplementary Table 1) resulted in the retention of 3 PA indexes and 1 sleep index: AEE, vigorous activity, steps, and WASO time. To ensure chance alone probabilities were held constant (50%) for each subgroup in analyses, separate classification models were generated to evaluate accuracies in identifying (1) OA vs control and (2) RA vs control condition membership from surviving SWA indexes. Because arthritis subgroups did not differ on SWA indexes, no OA versus RA model was generated.

Overall models were highly significant (*p* < .001) and indicated individual responses on included SWA indexes generated overall classification accuracy rates that exceeded chance levels, particularly for the identification of arthritis subgroup membership (see Table 4). In the RA versus CON model, 82.5% of adults with RA were correctly identified from the 4 SWA indexes. Step counts had a significant, unique impact within the classification model. The ROC analysis identified 9316 steps as the optimal cut-off value distinguishing groups (see Supplementary Fig. 1). In the OA versus CON LGA, 75% of OA patients were correctly identified from SWA index responses. Both steps and WASO made significant, unique contributions to the model. For these indexes, ROC analyses identified optimal cutoff values discriminating groups to be 9057 steps and a WASO time of 86 min (see Supplementary Fig. 2). Conversely, within each LGA, classification accuracies for control group members were attenuated albeit modestly higher than chance levels (< 60%) (Table 4).

**Table 4:** Accuracy of Sensewear Armband physical activity and sleep indexes in identifying each arthritis subgroup versus non-arthritic controls

	Group Classification Model			
	Rheumatoid Arthritis vs. Control		Osteoarthritis vs. Control	
**Sensewear Index**	Wald	p	Wald	p
Active energy expenditure	0.04	0.849	0.03	0.858
Vigorous activity	1.72	0.189	1.21	0.272
Steps (Mean number per day)	8.12	0.004	5.90	0.015
Wake after sleep onset time	0.67	0.413	4.27	0.039
**Groups**	Rheumatoid arthritis (N = 40)	Control (N = 40)	Osteoarthritis (N = 40)	Control (N = 40)
Arthritis subgroups (N = 40)	33/ 40 (82.5%)	7/ 40 (17.5%)	30/ 40 (75%)	10/ 40 (25%)
Control (N = 40)	14/ 40 (35.0%)	26/ 40 (65.0%)	13/ 40 (32.5%)	27/ 40 (67.5%)
Overall Model:	χ2 (4) = 24.007, *p* < 0.001		χ2 (4) = 20.541, p < 0.001	
	Total Correctly Classified: 73.8% (59/ 80)		Total Correctly Classified: 71.3% (57/ 80)	

## Discussion

Overall results underscored the superiority of objective measuresover self-report questionnaires in discriminating PA levels and sleep disturbances of adults with RA and OA compared to non-arthritic controls. First, with the exception of overall energy expenditures and lying down time, people with OA and RA differed significantly from controls on objective indexes tapping sedentary, moderate, and vigorous activity levels, active energy expenditures, overall activity levels > 3 METS, and PA durations. In contrast, estimates of time sitting down, walking, moderate activity, and vigorous activity based IPAQ-C responses did not discriminate between groups. The most glaring discrepancy was in relation vigorous activity. SWA data aligned with the hypothesis that non-arthritic controls would display elevations compared to each arthritis subgroup while questionnaire results indicated adults with OA reported over 5 h more of vigorous activity than controls did.

The pattern of group difference results dovetails with evidence of weaker validity of questionnaires than objective measures in differentiating PA levels of arthritis patients versus controls in Western samples [15, 16, 18]. Comparatively low average education levels may have contributed to the lack of group differences on IPAQ-C PA indexes. However, other researchers have argued biases in recall and social desirability as well as difficulties in mentally quantifying unstructured PA by frequency, intensity and duration also contribute to poor discriminant validity of PA questionnaires across studies [39].

Second, despite the absence of subgroup differences across SWA and questionnaire indexes of sleep onset, total sleep time and sleep efficiency, a notable discrepancy emerged for wake after sleep onset time (WASO). SWA data collected during sleep indicated arthritis subgroups, especially those with OA, displayed significantly more WASO than controls did. In contrast, self-reported estimates of WASO minutes collected following each nightly sleep were lowest among adults with OA and nearly 33% lower than estimates from controls. In related work, Roehrs et al. [5] identified more WASO time among patients with fibromyalgia and RA than pain-free controls during a nocturnal polysomnogram while subgroup differences were not evident on self-report sleep indexes. In tandem, these findings suggest WASO may be a key objective measure distinguishing sleep disturbances of various arthritis subgroups from controls while complementary questionnaire indexes have poor discriminant validity.

The utility of objective indexes in discriminating PA levels and sleep disturbances of arthritis subgroups versus controls was reinforced further by classification analysis results. Specifically, arthritis subgroup members, particularly those with RA, could be distinguished from controls at levels well above chance based on a subset of four SWA indexes. Although self-report indexes were excluded from subgroup classification analyses due to the complete absence of subgroup differences, objective assessments indicated RA and OA are characterized by specific deficits in PA and/or sleep compared to controls. Comparatively weaker classification accuracy levels of controls may have been a partial reflection of their generally greater variability in PA levels and sleep disruptions. This point is underlined by the typically larger standard deviations and wider individual differences on SWA indexes for controls illustrated in Table 2. Although chronic pain was an exclusion criterion in the selection of controls, per the general population, control group members may have shown greater heterogeneity in health, illness, and functioning than did cohorts experiencing limitations from arthritis.

The emergence of steps as the only PA index to discriminate both arthritis subgroups from controls in classification analyses is consistent with results from other arthritis research [18]. Indeed, decreased sedentary activity and increased light intensity activity – not just bouts of moderate to vigorous activity bouts – confer health benefits for arthritis groups [11]. Practically, then, step counts monitored via pedometers or simple mobile phone apps offer useful, inexpensive, objective PA measures for arthritis patients in China and abroad that are preferable to the IPAQ-C or other questionnaires susceptible to biases in reporting and recall.

Finally, in contrast to arthritis versus control differences on SWA indexes, no arthritis subgroup differences were found. This finding aligns with results of smaller N studies comparing different patient subgroups on objective measures of PA [15] and sleep [5]. The current sample was at least double the size of those from these studies so null effects were less likely to be a function of reduced statistical power. Even though OA and RA differ in prevalence, causes, courses, prognoses, and treatment [40–43], the lack of arthritis subgroup differences in reported interference from pain was at least partially attributable to the absence of corresponding differences on objective PA and sleep indexes. Furthermore, because ambulatory independence was a necessary selection criterion and groups did not differ on age, pain duration, or reported interference with daily activities due to pain, RA subgroup members may have been higher functioning than the population from which they were drawn.

Notwithstanding its implications, select limitations of this study warrant mention. First, samples were non-randomly selected Chinese community dwellers so caution is warranted in generalizing results to inpatients and those incapable of independent ambulation, other arthritis subtypes or groups in other countries. Second, even though the IPAQ-C and PSD discriminated poorly between the groups under study, this conclusion does not necessarily extend to all other self-report measures of PA and sleep. Finally, a small number of participants (*N* = 5) reported interference with sleep onset during their first night wearing the SWA and/or mild discomfort (slight redness, itching) with wearing the SWA for an extended period of 6–7 days. Fortunately, there was no evidence that adverse reactions differed between groups, confounded data collection, or continued after the armband was removed.

## Conclusion

This study indicated objective measures are preferable to specific questionnaires in discriminating experiences of PA and sleep among ambulatory Chinese adults with RA or OA compared to non-arthritic controls. However, converging with smaller N studies, arthritis subtype differences in objectively-assessed PA and sleep were not observed. Finally, classification analysis results underscored step counts as an easily available, cost-efficient and useful objective alternative to questionnaires in discriminating PA of arthritis subgroups versus controls.

## Supplementary Information

**Additional file 1.**

**Additional file 2.**

**Additional file 3.**

## Data Availability

The datasets used and/or analyzed during the current study are available from the corresponding author on reasonable request.

## Abbreviations

ANOVAs: Analyses of variance
AEE: active energy expenditure
BMI: body mass index
CON: controls
FNA: frequency of nightly awakenings
HRQoL: health related quality of life
IPAQ-SF-C: International Physical Activity Questionnaire Short Form-Chinese
LGA: logistic regression analyses
MANOVAs: Multivariate analyses of variance
METs: metabolic equivalents
OA: osteoarthritis
PA: physical activity
PAD: physical activity duration
PSD: Pittsburgh Sleep Diary
RA: rheumatoid arthritis
SE: sleep efficiency
SOL: sleep onset latency
SQ: sleep quality
SWA: Sensewear Armband
TIB: total time in bed
TST: total sleep time
WASO: waking after sleep onset
