# Supplementary material for: Physical activity and sleep differences between osteoarthritis, rheumatoid arthritis and non-arthritic people in China: objective versus self report comparisons
Source: BMC Public Health. 2021 Oct 9;21:1821. doi: 10.1186/s12889-021-11837-y (PMC8501529; doi:10.1186/s12889-021-11837-y)
Supplement: Supplementary file 1 — Additional file 1. [file 12889_2021_11837_MOESM1_ESM.docx]

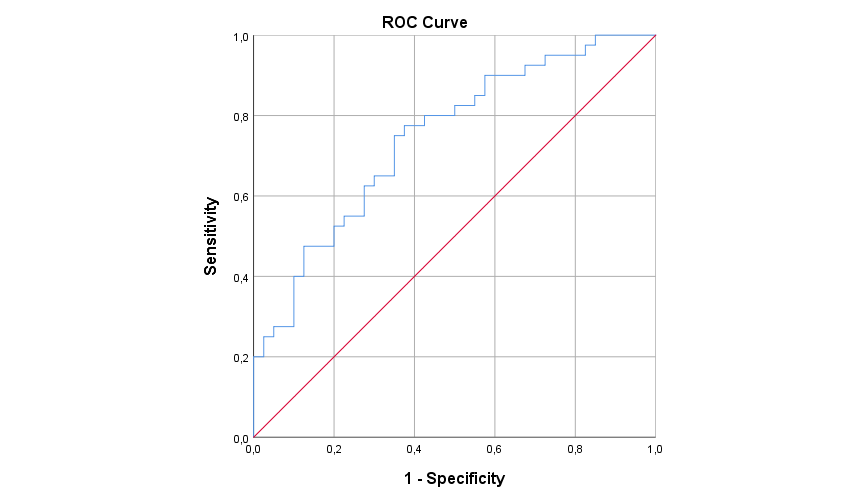


Supplementary figure 1. ROC Curve of steps to RA patients

**Supplementary table S1** Area under the curve

| Area | SD | P | Lower Bound (95% CI) | Upper Bound (95% CI) |
| --- | --- | --- | --- | --- |
| .745 | .054 | .001 | .639 | .851 |

RA: rheumatoid arthritis

*The greater the area under the curve, the greater the Steps ability to distinguish patients with arthritis. Youden's index (= sensitivity + 1 – specificity) was used as a criterion for selecting the optimum cut-off point in ROC curve.

Reference:

Hajian-Tilaki K. The choice of methods in determining the optimal cut-off value for quantitative diagnostic test evaluation. Statistical methods in medical research. 2018 Aug;27(8):2374-83.
