# Supplementary material for: Physical activity and sleep differences between osteoarthritis, rheumatoid arthritis and non-arthritic people in China: objective versus self report comparisons
Source: BMC Public Health. 2021 Oct 9;21:1821. doi: 10.1186/s12889-021-11837-y (PMC8501529; doi:10.1186/s12889-021-11837-y)
Supplement: Supplementary file 2 — Additional file 2. [file 12889_2021_11837_MOESM2_ESM.docx]

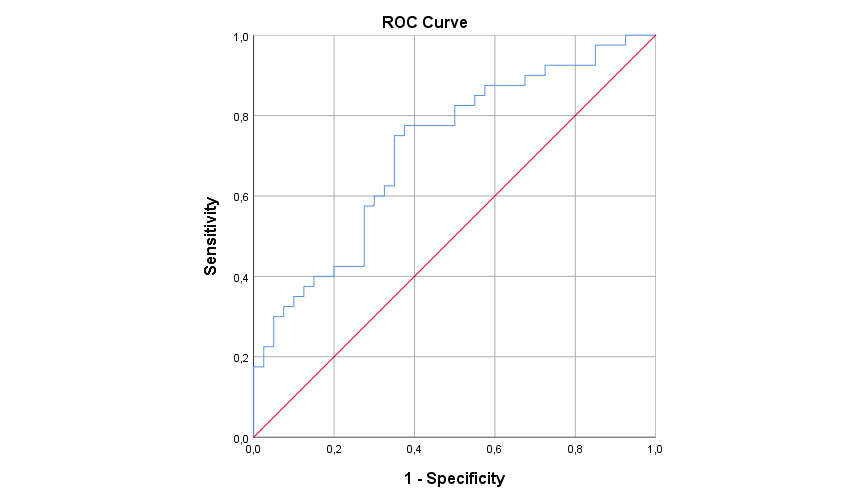


Figure 2.1. ROC Curve of Steps to OA patients

**Table2.1** Area under the curve

| Area | SD | P | Lower Bound (95% CI) | Upper Bound (95% CI) |
| --- | --- | --- | --- | --- |
| .715 | .057 | .001 | .603 | .827 |

OA: osteoarthritis


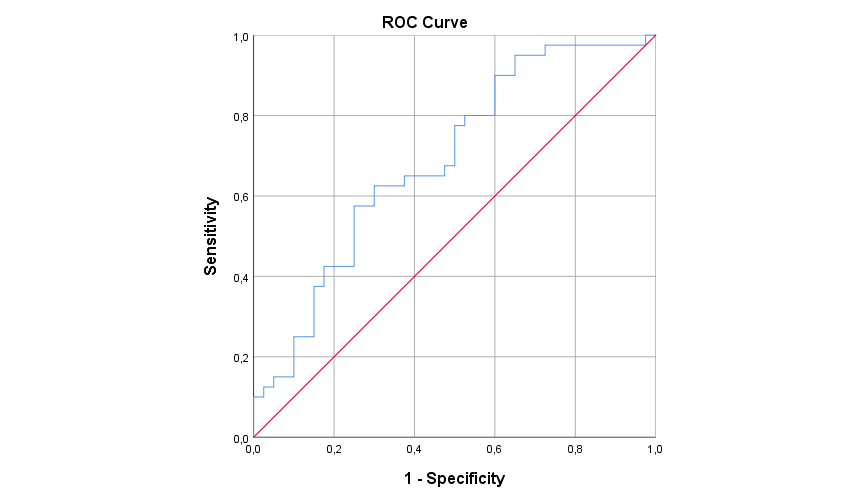


Figure 2.2. ROC Curve of WAS0 time to OA patients

**Table2.1** Area under the curve

| Area | SD | P | Lower Bound (95% CI) | Upper Bound (95% CI) |
| --- | --- | --- | --- | --- |
| .689 | .059 | .004 | .573 | .805 |

WAS0: time awake after sleep onset
