# Supplementary material for: Physical activity and sleep differences between osteoarthritis, rheumatoid arthritis and non-arthritic people in China: objective versus self report comparisons
Source: BMC Public Health. 2021 Oct 9;21:1821. doi: 10.1186/s12889-021-11837-y (PMC8501529; doi:10.1186/s12889-021-11837-y)
Supplement: Supplementary file 3 — Additional file 3. [file 12889_2021_11837_MOESM3_ESM.docx]

**Supplementary Table 1.** The model of the regression analysis with all PA indexes as the independent variables and WASO time as dependent variable (N = 120)

| Model | Standardized coefficients Beta | t | P | Collinearity tolerance | Statistic VIF |
| --- | --- | --- | --- | --- | --- |
| Sedentary activity | -0.03 | -0.05 | 0.96 | 0.02 | 43.50 |
| Moderate activity | -1.73 | -2.05 | 0.04 | 0.01 | 89.42 |
| Vigorous activity | -0.29 | -1.52 | 0.13 | 0.22 | 4.46 |
| Active energy expenditure | -0.16 | -1.14 | 0.26 | 0.41 | 2.41 |
| Physical activity level (METs) | -0.34 | -1.28 | 0.20 | 0.11 | 9.07 |
| Physical activity duration | 2.2 | 2.47 | 0.02 | 0.01 | 102.62 |
| Steps | -.023 | -2.14 | 0.03 | 0.67 | 1.50 |
